# Genome-Wide Analysis of the DUF4228 Family in Soybean and Functional Identification of *GmDUF4228–70* in Response to Drought and Salt Stresses

**DOI:** 10.3389/fpls.2021.628299

**Published:** 2021-05-17

**Authors:** Zhi-Xin Leng, Ying Liu, Zhan-Yu Chen, Jun Guo, Jun Chen, Yong-Bin Zhou, Ming Chen, You-Zhi Ma, Zhao-Shi Xu, Xi-Yan Cui

**Affiliations:** ^1^College of Life Sciences/College of Agronomy, Jilin Agricultural University, Changchun, China; ^2^National Key Facility for Crop Gene Resources and Genetic Improvement, Key Laboratory of Biology and Genetic Improvement of Triticeae Crops, Ministry of Agriculture, Institute of Crop Science, Chinese Academy of Agricultural Sciences (CAAS), Beijing, China; ^3^State Key Laboratory of Crop Stress Biology for Arid Areas, College of Plant Protection, Northwest A&F University, Yangling, China

**Keywords:** soybean, genome-wide analysis, *GmDUF4228* family, stress response mechanism, transgenic hairy root

## Abstract

Domain of unknown function 4228 (DUF4228) proteins are a class of proteins widely found in plants, playing an important role in response to abiotic stresses. However, studies on the DUF4228 family in soybean (*Glycine max* L.) are sparse. In this study, we identified a total of 81 *DUF4228* genes in soybean genome, named systematically based on their chromosome distributions. Results showed that these genes were unevenly distributed on the 20 chromosomes of soybean. The predicted soybean DUF4228 proteins were identified in three groups (Groups I–III) based on a maximum likelihood phylogenetic tree. Genetic structure analysis showed that most of the *GmDUF4228* genes contained no introns. Expression profiling showed that *GmDUF4228* genes were widely expressed in different organs and tissues in soybean. RNA-seq data were used to characterize the expression profiles of *GmDUF4228* genes under the treatments of drought and salt stresses, with nine genes showing significant up-regulation under both drought and salt stress further functionally verified by promoter (*cis*-acting elements) analysis and quantitative real-time PCR (qRT-PCR). Due to its upregulation under drought and salt stresses based on both RNA-seq and qRT-PCR analyses, *GmDUF4228-70* was selected for further functional analysis in transgenic plants. Under drought stress, the degree of leaf curling and wilting of the *GmDUF4228-70*-overexpressing (*GmDUF4228-70*-OE) line was lower than that of the empty vector (EV) line. *GmDUF4228-70*-OE lines also showed increased proline content, relative water content (RWC), and chlorophyll content, and decreased contents of malondialdehyde (MDA), H_2_O_2_, and O^2–^. Under salt stress, the changes in phenotypic and physiological indicators of transgenic plants were the same as those under drought stress. In addition, overexpression of the *GmDUF4228-70* gene promoted the expression of marker genes under both drought and salt stresses. Taken together, the results indicated that *GmDUF4228* genes play important roles in response to abiotic stresses in soybean.

## Introduction

Abiotic stresses are the main limiting factors affecting plant growth and yield, causing significant agricultural and economic losses for farmers worldwide (Ahmad et al., 2014; Sade et al., 2018). For example, 70% of the total annual loss of yield potential is estimated to be due to the imbalance of the physical and chemical environments (Boyer, 1982). Drought and salt stresses are the main abiotic factors affecting the geographical distribution of plants, limiting the crop yield, and threatening food security (Mahalingam and Fedoroff, 2010). In general, plants lack the structures directly functioning in environmental perception, but could respond similarly to environmental changes (Hamant and Haswell, 2017). Plants show extensive defensive responses at the molecular and cellular levels to resist damage to cells caused by stresses (Kim et al., 2014).

Soybean (*Glycine max* L.) is a major agricultural crop, widely used as food source for humans and livestock due to its rich contents of oil, proteins, and minerals (Papiernik et al., 2005). It is also processed to produce traditional healthy foods in Chinese culture because of its nutritional composition and pharmacological value (Wang and Komatsu, 2018). Legume crops are suitable for growing in a wide range of climatic conditions, though they are extremely sensitive to flooding, drought, and salt (Hossain et al., 2013), especially during its seedling development and early reproductive stages (Gupta et al., 2020; Zhao et al., 2020). Under stress conditions, transcription factors such as bZIP and NAC form a complex regulatory network by binding to specific *cis*-acting elements and activating stress responsive genes, ultimately enabling plants to resist external stresses (Zong et al., 2016; Ma et al., 2018). The identification of genes that increase drought and salt tolerance of crops is essential for the effective agricultural use of land (Guan et al., 2014) and is of far-reaching significance of broadening the genetic basis of soybeans, enhancing their resistance to stress, and ensuring their sustainable production.

Domain of unknown function (DUF) proteins contain at least one highly conserved DUF domain and are widely found in plants (Bateman et al., 2010). Currently, there are more than 370,0 DUFs listed in the Pfam database, accounting for ∼25% of known domains (Finn et al., 2008). DUF1 and DUF2 were added to the Pfam database by Chris Ponting, who named the DUF domain and proposed naming these domains using a combination of “DUF” and a number (Schultz et al., 1998). Systematic structure analysis clarified that many DUFs may have arisen from the extreme diversification and neofunctionalization of known protein domains (Jaroszewski et al., 2009).

DUF4228 proteins are members of the DUF superfamily. Studies have shown that DUF4228 family members play an important role in response to abiotic stress in plants (Qi et al., 2019). Three members of DUF4228 family in *Arabidopsis thaliana*, i.e., *AT1G10530*, *AT1G21010*, and *AT1G28190*, are involved in response to drought stress (Yang Q. et al., 2020). Overexpression of the *Medicago sativa DUF* (*MsDUF*) gene in tobacco resulted in significantly lowered chlorophyll and soluble sugar contents and increased malondialdehyde (MDA) content. Expression of the *MsDUF* gene was significantly decreased under the treatments of NaCl, PEG6000, abscisic acid (ABA), and gibberellic acid (GA), indicating that *MsDUF* played a negative regulatory role in stress resistance of *Medicago sativa* (Wang et al., 2017). Expression of the *Caragana intermedia DUF4228-3* (*CiDUF4228-3*) gene was significantly up-regulated under dehydration, low temperature, and drought, indicating its involvement in stress response (Na et al., 2016). Recent studies revealed that *DUF4228* genes were involved in abiotic stress response and cadmium tolerance. Analysis of *AtDUF4228* gene expression under a variety of stress treatments and co-expression network analysis of *DUF4228* genes in different species of plants showed that several *DUF4228* genes may play a synergistic role in plant defense (Didelon et al., 2020). These results suggest that members of the DUF4228 family may respond to abiotic stresses in soybean.

Although the whole genome of soybean was sequenced 10 years ago, studies investigating the *GmDUF4228* gene family in soybean are still lacking. In this study, we identified a total of 81 *GmDUF4228* genes and further analyzed their phylogenetic relationships, protein characteristics, gene structures, motifs, promoters, and expression patterns in various tissues and organs under different stress conditions. The expression levels of *GmDUF4228-13*, *–43*, *–45*, *–48*, *–51*, *–58*, *–61*, *–70*, and *–80* under drought and salt stresses were analyzed by quantitative real-time PCR (qRT-PCR), and the function of the *GmDUF4228-70* gene under drought and salt stresses was investigated. The findings provide a scientific foundation for further studies of the functions of *GmDUF4228* genes.

## Materials and Methods

### Identification of DUF4228 Genes in Soybean

The Hidden Markov Model (HMM) corresponding to the DUF4228 family (PF14009) was used to identify DUF4228 proteins in the soybean genome using HMMER 3.0. Proteins with an *E*-value greater than 1e-20 were excluded for further analysis. All qualified GmDUF4228 protein sequences were extracted and aligned to build a soybean-specific HMM profile using the hmmbuild tool in HMMER 3.0. The new HMM profile was used to search a local protein database. Proteins with an *E*-value less than 0.001 were retained. After removing redundant sequences, the remaining protein sequences were submitted to the SMART^1^ and NCBI batch CD-search databasesT^2^ to confirm the presence of the DUF4228 domain (Shan et al., 2020).

### Phylogenetic Analysis, Protein Characteristics, Gene Structure, and Motif Analysis of GmDUF4228

The amino acid sequences of DUF4228s derived from *Arabidopsis* and *Medicago truncatula* combined with newly identified GmDUF4228s in this study were used to construct phylogenetic trees. Multiple sequence alignment was conducted using the E-INS-I option of MAFFT 7.0 (Katoh and Standley, 2013). Gblocks 0.91b was used to remove ambiguous regions in the alignment with conserved regions used to construct the phylogenetic trees (Castresana, 2000). The maximum likelihood (ML) phylogenetic trees of a total of 135 DUF4228 protein sequences of *Arabidopsis*, *Medicago truncatula*, and soybean were reconstructed using MEGA 7.0 based on the JTT + G model with a 4-categories GAMMA distribution. The JTT + G model was identified based on ProTest (Posada, 2011). The bootstrap analysis was conducted with 1,000 replicates. To cluster the genetic structure, motifs, and expression profiles of GmDUF4228 genes in various tissues and organs of soybean, the maximum likelihood (ML) phylogenetic trees of a total of 81 DUF4228 protein sequences of only soybean were reconstructed using MEGA 7.0 based on the JTT + I + G model with a 4-categories GAMMA distribution. The JTT + I + G model was identified based on ProTest. The bootstrap analysis was conducted with 1,000 replicates. ExPASYT^3^ was used to calculate the molecular weight (MW) and isoelectric point (p*I*) of each GmDUF4228 protein sequence. The coding sequence (CDS) and cDNA sequences corresponding to each *GmDUF4228* gene were submitted to the GSDS databaseT^4^ to analyze the exon-intron structure. The conserved motifs were analyzed using MEMET^5^.

### Chromosome Localization and Analysis of Potential *Cis*-Elements

The information of the chromosome locations of GmDUF4228 family was obtained from the Phytozome databaseT^6^. All *GmDUF4228* genes were mapped to the soybean chromosomes based on the genomic annotations. The 2,000 bp regions upstream of the start codon of 9 GmDUF4228 genes showing significant up-regulation in both drought and salt stresses were submitted to PlantCARET^7^ to identify the *cis*-acting elements and calculate the number of each element.

### Expression Patterns of *GmDUF4228* Genes in Different Tissues and Organs of Soybean

The fragments per kilobase of transcript per million mapped reads (FPKM) values for 81 GmDUF4228 family members in nine different tissues and organs (apical meristems, flowers, leaves, nodules, stems, pods, roots, root hairs, and seeds) under normal conditions were retrieved from the Phytozome database (see text footnote 6). TBtools software was used to draw a heatmap of expression data (Chen et al., 2020).

### Plant Materials and Growth Conditions

The seeds of soybean *Williams* 82 were planted in a 10 × 10 cm pot. The leaves of soybean seedlings grown in the greenhouse for 15 days (with a photoperiod cycle of 16 h light/8 h dark, 25°C and/20°C at day and night, respectively, and 60% relative humidity) were used for total RNA extraction and qRT-PCR analysis. For drought stress, seedlings were placed on filter paper. Samples were collected at 0, 0.5, 1, 2, 4, 8, 12, and 24 h after treatment. For salt stress, the roots of seedlings were placed in a 200 mM NaCl solution. Samples were also collected at 0, 0.5, 1, 2, 4, 8, 12, and 24 h after treatment (Su et al., 2020). The collected samples were quickly moved into liquid nitrogen and then stored at −80°C for later use.

### RNA Extraction and qRT-PCR

Total RNA was extracted from young plant samples using an RNA extraction and purification kit according to the manufacturer’s protocol (TIANGEN, Beijing, China) and reverse transcribed into cDNA using a PrimeScriptTM RT Reagent Kit (TaKaRa, Japan). The ABI prism 7500 sequence detection system (Applied Biosystems) was used for expression analysis based on qRT-PCR with three biological replicates for each sample. The 2^–ΔΔ^ ^*CT*^ method was used to calculate the relative changes in gene expression (Cui et al., 2018). The qRT-PCR primers used to amplify *GmDUF4228* genes were designed using Primer Premier 5.0 software. Soybean *actin* gene was used as the internal control for qRT-PCR (Zhao et al., 2017; Supplementary Table 1).

### Construction of the *GmDUF4228-70* Vector

The CDS of *GmDUF4228-70* was amplified from *Williams* 82 cDNA with gene-specific primers containing restriction site sequences for *Nco*I and *BsTE*II. The PCR products and the pCAMBIA3301 vector were digested with *Nco*I and *BsTE*II (Thermo Fisher Scientific, United States), and the products were ligated to obtain pCAMBIA3301-*GmDUF4228-70* (*GmDUF4228-70*-OE) lines (Kereszt et al., 2007).

### *Agrobacterium rhizogenes*-Mediated Transformation of Soybean Hairy Roots

*GmDUF4228-70*-OE was studied using soybean hair roots, generated from soybean seedlings using *Agrobacterium rhizogenes* K599, which contained either the pCAMBIA3301 empty vector (EV) or pCAMBIA3301-*GmDUF4228-70*-OE and was injected 2 mm below different cotyledonary nodes. When the new roots grew to 5 cm in length, the old roots were excised. After 5 days of cultivation, transgenic plants were prepared for stress treatment as described by Kereszt et al. (2007).

### Drought and Salt Stress Assays

After verification, the positive hairy roots of soybean were used in abiotic stress assays. Five transgenic soybean seedlings were cultivated in each pot with each stress treatment repeated for five times. For drought stress, the EV and *GmDUF4228-70*-OE plants were first dehydrated for 15 days, and then rewatered for 3 days. For salt stress, the EV and *GmDUF4228-70*-OE plants were treated with 200 mM NaCl for 7 days as described by Yang Y. et al. (2020).

### Relative Water Content and Chlorophyll Content

To determine the relative water content (RWC), fresh leaves of the EV and *GmDUF4228-70*-OE plants were weighed. The leaves were completely immersed in sterile water for 12 h and then removed. Absorbent paper was used to absorb the excess water on the surface of the leaves and leaves were weighed to obtain the saturated weight. Finally, the leaves were wrapped in tin foil and incubated at 65°C for more than 72 h. After the leaves completely lost moisture, they were weighed to obtain the dry weight. A Sartorius BSA224S-CW 1/10,000 analytical balance (Sartorius, Beijing, China) was used to weigh the samples (Lu et al., 2009). For determination of chlorophyll content, fresh leaves (0.1 g) from EV and *GmDUF4228-70*-OE plants were cut into strips and immediately placed in a mixture of 50% anhydrous ethanol and 50% acetone and incubated in dark for 12 h. Then, 200 μl extract was extracted from the centrifuge tubes containing different samples and added to the enzyme plates. The absorbance values at 645 nm and 663 nm were measured using the Varioskan LUX Multimode Microplate Reader (Thermo Fisher Scientific, Waltham, MA, United States). Each measurement was made from three biological replicates.

### Measurement of Proline, Malondialdehyde, H_2_O_2_, and O^2–^ Contents

The contents of proline, MDA, H_2_O_2_, and O^2–^ were measured with the corresponding assay kits (Cominbio, Suzhou, China) based on the manufacturer’s protocols. All measurements were made from three biological replicates.

### Trypan Blue, DAB, and NBT Staining

The transgenic soybean seedlings were cultivated without water for 7 days or treated with 200 mM NaCl solution for 3 days. The leaves of the EV and the *GmDUF4228-70*-OE seedlings were removed and were then submerged in 0.4% trypan blue, DAB (3,3′-diaminobenzidine) (Solarbio, Beijing, China), or NBT (nitroblue tetrazolium) (Solarbio, Beijing, China) staining solution for 12 h, respectively. Finally, the stained leaves were immersed in 75% ethanol until the leaves turned white.

## Results

### Genome-Wide Identification and Phylogenetic Analysis of GmDUF4228 Family Members

**FIGURE 1:**
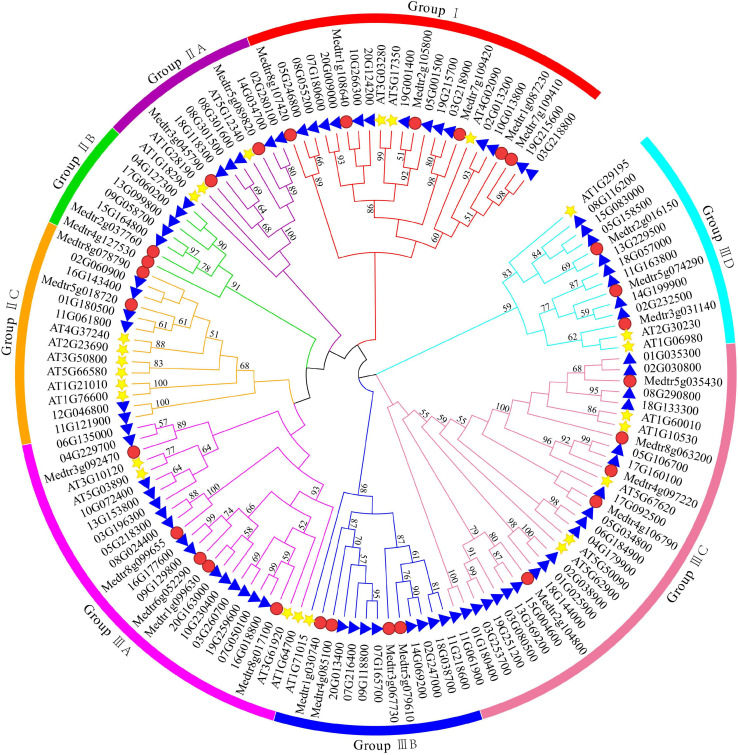
Phylogenetic tree of DUF4228 proteins from *Arabidopsis*, *Medicago truncatula*, and soybean using the ML method. The colored arcs indicate different groups. The stars, circles, and triangles represent proteins of *Arabidopsis Medicago truncatula*, and soybean, respectively.

In this study, a total of 81 DUF4228 family members were identified from the soybean genome database and were named *GmDUF4228-1* to *GmDUF4228-81* according to their chromosomal distributions (Su et al., 2019). A total of 135 amino acid sequences (i.e., 25 AtDUF4228 proteins, 29 MtDUF4228 proteins, and 81 GmDUF4228 proteins) were compared to evaluate their evolutionary relationships, and a phylogenetic tree was built in MEGA 7.0 using the ML method (Figure 1). The topology of the ML tree showed that the 135 GmDUF4228s were identified in three groups (Groups I to III). The predicted GmDUF4228 polypeptide sequences ranged from 141 to 659 amino acids in length, with MW ranging from 15.24 to 75.27 kDa, and p*I* ranging from 5.59 to 10.13 (Table 1).

**TABLE 1:** Molecular characteristics of the *GmDUF4228* genes identified in soybean.

Gene name	Gene ID	CDS (bp)	ORF (aa)	MW (kDa)	p*I*	Chromosome
*GmDUF4228-1*	01G025900	729	242	27.4	9.77	1
*GmDUF4228-2*	01G035300	519	172	19.14	10.11	1
*GmDUF4228-3*	01G180400	825	275	30	6.88	1
*GmDUF4228-4*	01G180500	480	159	16.96	8.37	1
*GmDUF4228-5*	02G013200	543	180	20.78	8.87	2
*GmDUF4228-6*	02G030800	519	172	19.19	10.11	2
*GmDUF4228-7*	02G038900	750	249	27.98	9.51	2
*GmDUF4228-8*	02G060900	498	165	17.96	8.27	2
*GmDUF4228-9*	02G232500	516	171	18.68	9.86	2
*GmDUF4228-10*	02G247000	645	214	23.62	9.74	2
*GmDUF4228-11*	02G280100	687	228	25.54	8.53	2
*GmDUF4228-12*	03G080500	804	267	30.93	9.8	3
*GmDUF4228-13*	03G196300	471	156	17.71	8.56	3
*GmDUF4228-14*	03G218800	600	199	22.72	8.98	3
*GmDUF4228-15*	03G218900	498	165	18.12	9.46	3
*GmDUF4228-16*	03G253700	729	242	27.63	10.08	3
*GmDUF4228-17*	03G260700	615	204	22.44	5.59	3
*GmDUF4228-18*	04G127300	426	141	15.24	7.73	4
*GmDUF4228-19*	04G179900	522	173	19.24	9.83	4
*GmDUF4228-20*	04G229700	447	148	16.24	9.32	4
*GmDUF4228-21*	05G001500	486	161	17.91	5.79	5
*GmDUF4228-22*	05G034800	543	180	20.06	9.51	5
*GmDUF4228-23*	05G106700	543	180	19.57	9.52	5
*GmDUF4228-24*	05G158500	528	175	19.51	9.94	5
*GmDUF4228-25*	05G218300	645	214	23.37	8.04	5
*GmDUF4228-26*	05G246800	504	167	19.14	6.91	5
*GmDUF4228-27*	06G135000	447	148	16.29	9.56	6
*GmDUF4228-28*	06G184900	546	181	20.21	9.94	6
*GmDUF4228-29*	07G050100	594	197	21.81	6.84	7
*GmDUF4228-30*	07G165700	621	206	22.57	9.87	7
*GmDUF4228-31*	07G180600	513	170	19.29	7.68	7
*GmDUF4228-32*	07G216400	633	210	23.55	9.73	7
*GmDUF4228-33*	08G024400	630	209	22.91	8.54	8
*GmDUF4228-34*	08G055200	507	168	19.29	6.9	8
*GmDUF4228-35*	08G116200	528	175	19.7	9.9	8
*GmDUF4228-36*	08G290800	531	176	19.65	9.81	8
*GmDUF4228-37*	08G301500	645	214	24.23	8.97	8
*GmDUF4228-38*	08G301600	651	216	24.12	9.14	8
*GmDUF4228-39*	09G058700	546	181	19.79	9.54	9
*GmDUF4228-40*	09G118800	600	199	21.6	9.71	9
*GmDUF4228-41*	09G129800	741	246	26.46	8.56	9
*GmDUF4228-42*	10G013800	603	200	22.7	7.74	10
*GmDUF4228-43*	10G072400	489	162	18.13	8.82	10
*GmDUF4228-44*	10G230400	544	247	26.74	6.29	10
*GmDUF4228-45*	10G266300	543	180	20.27	8.79	10
*GmDUF4228-46*	11G061800	492	163	17.76	8.73	11
*GmDUF4228-47*	11G061900	444	147	15.69	7.68	11
*GmDUF4228-48*	11G121900	435	144	15.65	9.1	11
*GmDUF4228-49*	11G163800	504	167	18.32	9.64	11
*GmDUF4228-50*	11G218600	645	214	23.69	9.79	11
*GmDUF4228-51*	12G046800	432	143	15.63	8.91	12
*GmDUF4228-52*	13G099800	516	171	18.69	8.62	13
*GmDUF4228-53*	13G153800	486	161	18.07	9.28	13
*GmDUF4228-54*	13G229500	588	195	21.9	9.11	13
*GmDUF4228-55*	13G369200	753	250	27.93	9.47	13
*GmDUF4228-56*	14G034700	660	219	24.3	8.63	14
*GmDUF4228-57*	14G069200	645	214	23.54	9.6	14
*GmDUF4228-58*	14G199900	504	167	18.69	9.37	14
*GmDUF4228-59*	15G004600	768	255	28.49	8.98	15
*GmDUF4228-60*	15G083000	531	176	19.71	9.83	15
*GmDUF4228-61*	15G164800	537	178	19.52	9.57	15
*GmDUF4228-62*	16G018800	600	199	22.04	6.17	16
*GmDUF4228-63*	16G143400	516	171	18.33	8.71	16
*GmDUF4228-64*	16G177600	735	244	26.58	9.11	16
*GmDUF4228-65*	17G060200	513	170	18.48	8.93	17
*GmDUF4228-66*	17G092500	549	182	20.15	9.75	17
*GmDUF4228-67*	17G160100	537	178	19.51	9.73	17
*GmDUF4228-68*	18G038700	657	218	24.26	9.83	18
*GmDUF4228-69*	18G057000	498	165	18.09	9.74	18
*GmDUF4228-70*	18G118300	594	197	22.09	8.71	18
*GmDUF4228-71*	18G133300	525	174	19.2	9.91	18
*GmDUF4228-72*	18G144000	1980	659	75.27	8.79	18
*GmDUF4228-73*	19G001400	489	162	18.16	6.84	19
*GmDUF4228-74*	19G215600	600	199	22.79	8.96	19
*GmDUF4228-75*	19G215700	441	146	16.05	9	19
*GmDUF4228-76*	19G251200	678	225	25.88	10.13	19
*GmDUF4228-77*	19G259600	645	214	23.71	6.45	19
*GmDUF4228-78*	20G009000	513	170	19.1	5.95	20
*GmDUF4228-79*	20G013400	639	212	23.47	9.59	20
*GmDUF4228-80*	20G124200	534	177	19.67	8.96	20
*GmDUF4228-81*	20G163000	708	235	25.41	6.75	20

**FIGURE 2:**
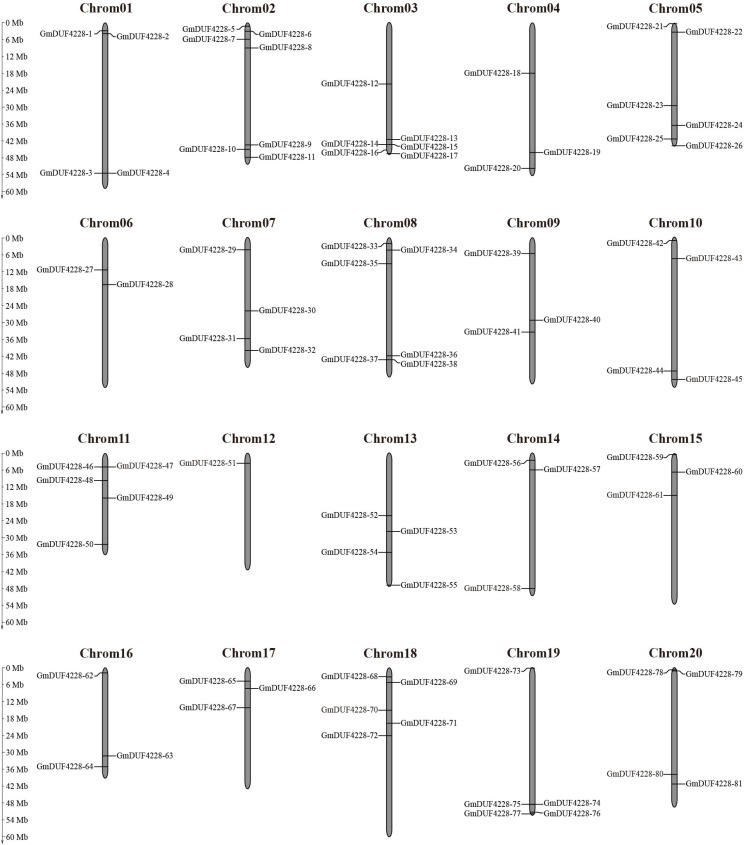
Chromosomal distribution of *GmDUF4228* genes. The scale bar indicates the length (Mb) of soybean chromosomes.

The 81 *GmDUF4228* genes were unevenly distributed on the twenty chromosomes of soybean with chromosome 2 containing the largest number of genes (7) (Figure 2). Six genes were located on chromosomes 3, 5, and 8, respectively, while only one gene (*GmDUF4228-51*) was found on chromosome 12. Each of other chromosomes contained 2–5 genes.

### Structure and Motif Analyses of *GmDUF4228* Genes

**FIGURE 3:**
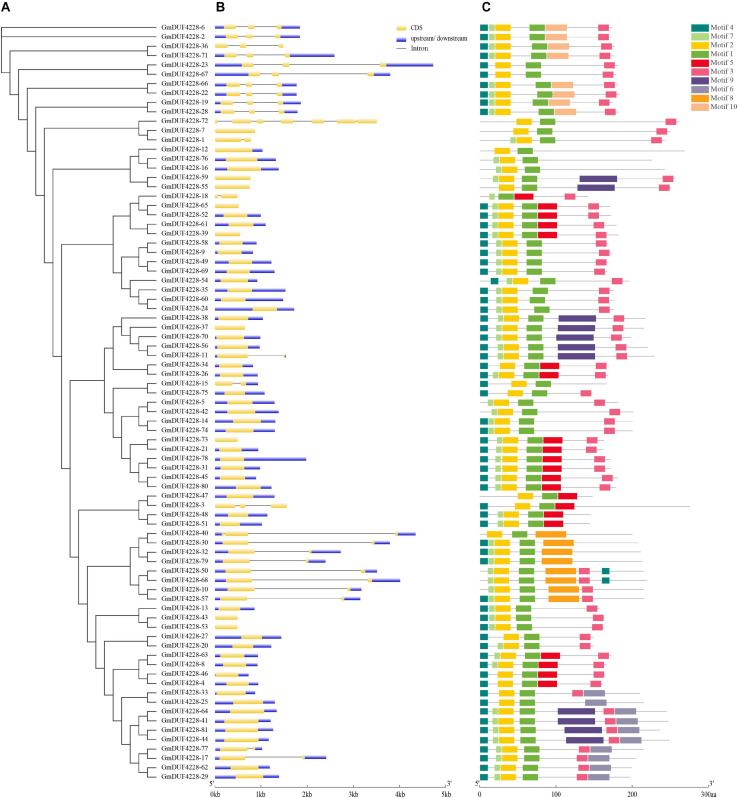
Phylogenetic relationships, gene structures, and conserved motifs of GmDUF4228. **(A)** Phylogenetic tree of DUF4228 proteins from soybean constructed using the ML method. **(B)** Exon/intron organization of *GmDUF4228* genes. Yellow boxes represent exons and black lines represent introns. The upstream/downstream regions of *GmDUF4228* genes are indicated by blue boxes. The scale at the bottom is used to infer the lengthes of exons. **(C)** Distribution of conserved motifs in *GmDUF4228* genes. Ten putative motifs are indicated by colored boxes (Table 2).

The phylogenetic tree based on the 81 amino acid sequences of GmDUF4228 proteins was constructed using ML method. The gene structure and motifs were clustered according to the ML phylogenetic tree (Figure 3A). Diversification of gene structure plays an important role in gene family evolution (Schmutz et al., 2010). Our results showed that most *GmDUF4228* genes contained no or 1–2 introns with *GmDUF4228-72* containing 7 introns (Figure 3B). Specifically, no intron was detected in 55 *GmDUF4228* genes (68%), while 1 and 2 introns were identified in 13 (16%) and 12 *GmDUF4228* genes (15%), respectively.

Amino acid sequences were analyzed using MEME in order to further characterize the structure of the GmDUF4228 proteins (Figure 3C). A total of 10 conserved motifs ranging in length from 8 to 50 amino acids were identified (Table 2).

**TABLE 2:** List of the putative motifs in GmDUF4228 proteins.

Motif number	Length (aa)	Best possible match
1	21	RIKPLPPDEELQPGQVYFLLP
2	21	VEELKTPITASEVMKENPGHV
3	15	KSRVWKPSLETISEE
4	11	MGNCLSCDLAT
5	26	MSRLNSPVSPVDMAALAVKATSAAKK
6	29	LLEILSQEARTEALIESVRTVAKCGNGAP
7	8	KVVHPBGK
8	41	RVRGGRGMNAKDRLESLVLSKRSVSDLTJMK QGPNPENDGP
9	50	SVPNKSTIKFGRCSFEYLKGSNGRVLIK VSPEFITRLISRSKRPCCNSSN
10	29	TQEVMKGLKAKKHAKMRKPLAESAZKPNL

### Expression Profiles of the *GmDUF4228* Genes in Various Tissues and Organs of Soybean

**FIGURE 4:**
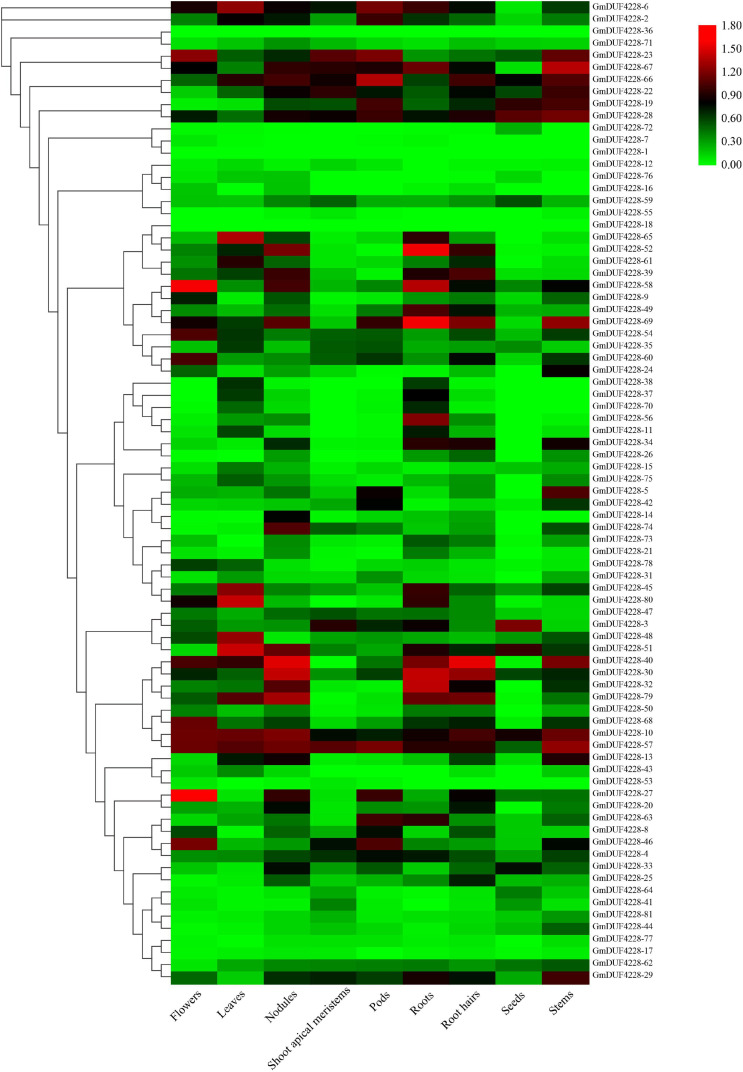
Expression heatmap of *GmDUF4228* genes in different tissues and organs of soybean constructed with TBtools. Fragments per kilobase of transcript per million mapped reads (FPKM) values of *GmDUF4228* genes are transformed by log10. Red and green represent high and low expressions, respectively. Genes are ordered according to the phylogenetic tree of soybean DUF4228 proteins shown in Figure 3.

In order to explore the expression pattern of *GmDUF4228* genes in different tissues and organs during soybean development, we extracted the transcriptome data of *GmDUF4228* genes from nine soybean tissues and organs, including apical meristems, flowers, leaves, nodules, stems, pods, roots, root hairs, and seeds, from the publically available RNA-seq data in Phytozome database. We also used the amino acid sequences of 81 GmDUF4228 proteins to construct a phylogenetic tree using ML method to cluster the expression profiles of soybean *GmDUF4228* genes in different tissues and organs (Figure 4).

Results showed that *GmDUF4228-10* and *–46* showed high expression levels in all nine tissues and organs of soybean, while some genes were highly expressed in only one tissue. For example, *GmDUF4228-5*, *–14*, *–26*, and *–75* showed high expression levels in pods, nodules, root hairs, and leaves, respectively, while *GmDUF4228-56*, *–70*, and *–73* were expressed mainly in roots. These transcription profiles suggested that *GmDUF4228* genes may be involved in the development of soybean plants.

### Responses of *GmDUF4228* Genes to Drought and Salt Treatments

**FIGURE 5:**
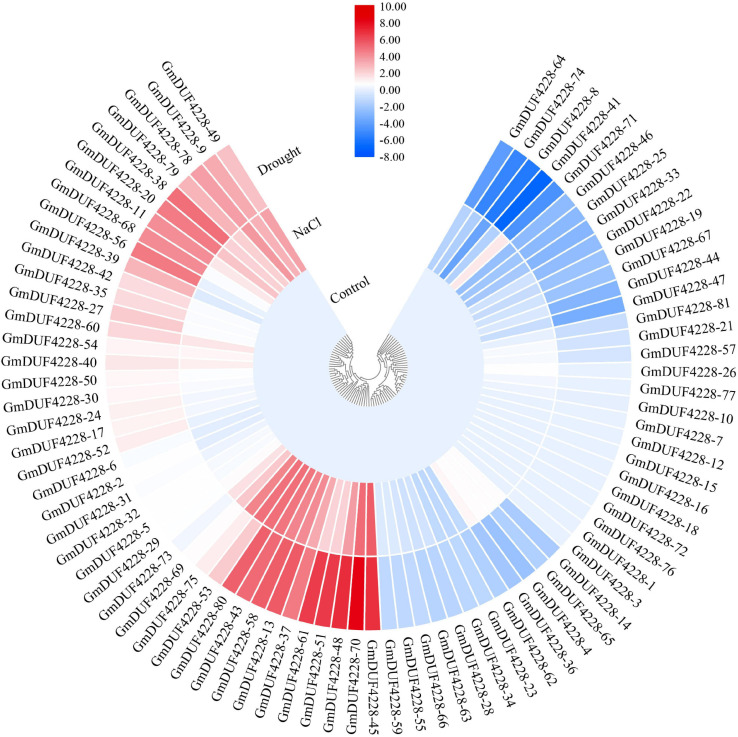
Heatmap based on hierarchical cluster analysis of 81 *GmDUF4228* gene expression profiles under drought and salt stresses and control conditions. Red and blue represent high and low expressions, respectively.

To investigate the potential functions of *GmDUF4228* genes under abiotic stress, we examined the transcription levels of *GmDUF4228* genes under drought and salt stresses based on previously published RNA-seq data (Shi et al., 2018). The results showed that most of the *GmDUF4228* genes were induced by drought and salt (Figure 5). A total of 22 genes were up-regulated under drought stress (based on the criterion of “fold change ≥ 2”), such as *GmDUF4228-45*,–*48*, –*51*, –*61*, and –*70*, while 15 genes were down-regulated (based on the criterion of “fold change ≤ 2”), such as *GmDUF4228*-8, –41, –64, –71, and –74. A total of 18 genes were up-regulated under salt stress, such as *GmDUF4228-43*, –*45*, –*53*, –*70*, and –*80*, and 4 genes were down-regulated, including *GmDUF4228-8*, *−25*, *−33*, and *–46*. It was worth mentioning that four genes (i.e., *GmDUF4228-4*, *–36*, *–39*, and *–71*) showed opposite expression patterns between the treatments of drought and salt stresses. To further confirm the expression of *GmDUF4228* genes under abiotic stresses, we selected nine genes (*GmDUF4228-13*, *–43*, *8-45*, *–48*, *–51*, *–58*, *–61*, *–70*, and *–80*) significantly up-regulated under both drought and salt stresses for further functional verification based on promoter (*cis*-acting element) analysis and gene expression analysis using qRT-PCR.

### Analysis of *Cis*-Acting Elements in *GmDUF4228* Promoters

**FIGURE 6:**
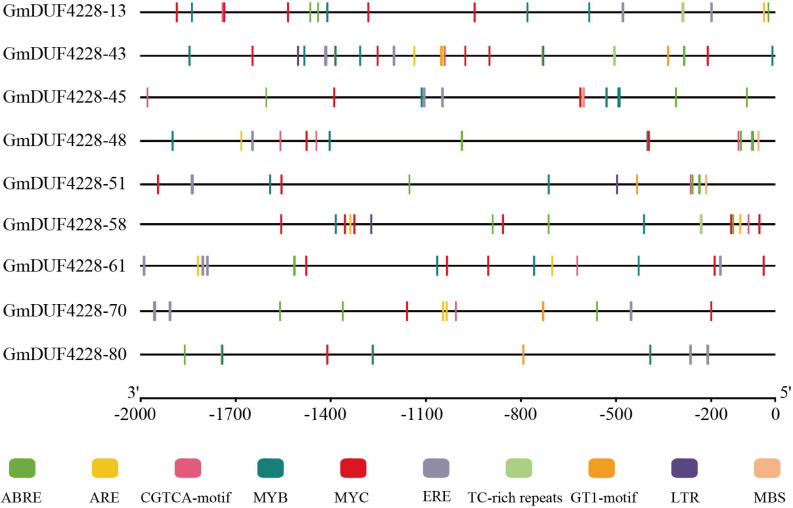
Predicted *cis*-acting elements in *GmDUF4228* promoters. Promoter sequences (2,000 bp) of nine *GmDUF4228* genes were analyzed by PlantCARE. The scale at the bottom is used to infer the length of upstream sequences starting from the starting site of translation.

The 2,000 bp regions upstream of the start codon in the nine *GmDUF4228* genes were analyzed for *cis*-acting elements (Figure 6). Ten elements related to abiotic stress were identified to further investigate the role of these nine genes in response to abiotic stresses. These elements included the ABA-responsive element (ABRE), anaerobic induction element (ARE), methyl jasmonate-responsive element (CGTCA-motif), ethylene-responsive element (ERE), low temperature response element (LTR), three drought and salt-responsive elements (MYB, MYC, and MBS), defense and stress responsive elements (TC-rich repeats), and light-responsive element (GT1-motif). Results showed that each gene promoter contained at least five or more *cis*-acting elements related to abiotic stress, with five *cis*-acting elements (i.e., the ABRE, CGTCA-motif, MYB, MYC, and ERE) revealed in most of the *GmDUF4228* gene promoter regions. These results indicated that these nine candidate *GmDUF4228* genes might be involved in response to abiotic stresses.

### qRT-PCR Analysis of *GmDUF4228* Genes

**FIGURE 7:**
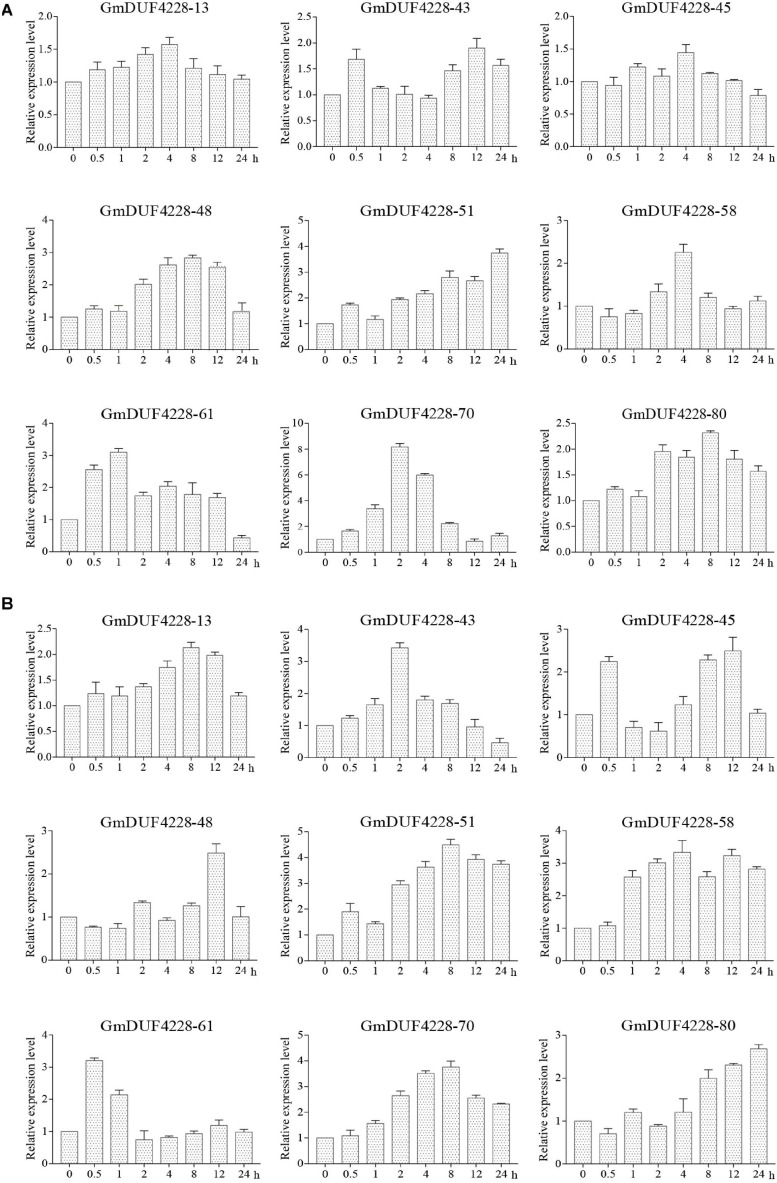
Expression patterns based on qRT-PCR of nine *GmDUF4228* genes under drought and salt stresses. **(A)** Drought treatment under 0, 0.5, 1, 2, 4, 8, 12, and 24 h. **(B)** Salt treatment (200 mM NaCl) for 0, 0.5, 1, 2, 4, 8, 12, and 24 h. Data are presented as mean ± standard deviation (*n* = 3).

To investigate the potential functions of the nine *GmDUF4228* genes in response to different stimuli, qRT-PCR was used to analyze the expression patterns of these genes in plants treated under drought and salt stresses (Figure 7). Under drought stress (Figure 7A), the expression levels of *GmDUF4228-48* and *–80* reached their peaks at 8 h and those of *GmDUF4228-51*, *–58*, *–61*, and *–70* peaked at 24, 4, 1, and 2 h (> 2-fold), respectively, while *GmDUF4228-70* showed expression level more than seven times higher than that of the control. Under salt stress, the expression levels of genes *GmDUF4228-13*, *-45*, *–48*, *–51*, and *–70* reached their peaks at 8–12 h, while those of *GmDUF4228-43*, *–58*, *–61*, and *–80* peaked at 2, 4, 0.5, and 24 h, respectively (Figure 7B). These results indicated that the transcription levels of most of these nine *GmDUF4228* genes were affected by drought and salt stresses. *GmDUF4228-70* was selected for further functional studies due to its relatively evident transcription changes under both drought and salt stresses.

### Overexpression of *GmDUF4228-70* Improved Drought and Salt Tolerance

To study the biological function of *GmDUF4228*-*70* in soybean, a *GmDUF4228-70* overexpression vector (*GmDUF4228-70*-OE) and empty vector (EV) were transferred into soybean hairy roots by *Agrobacterium*-mediated transformation, and the phenotypes of seedlings under drought and salt stresses were evaluated. Results of the qRT-PCR analysis showed that the *GmDUF4228-70* transcripts in the hairy roots of *GmDUF4228-70*-OE soybean seedlings were significantly higher than those in seedlings of EV control under normal conditions (Supplementary Figure 1).

**FIGURE 8:**
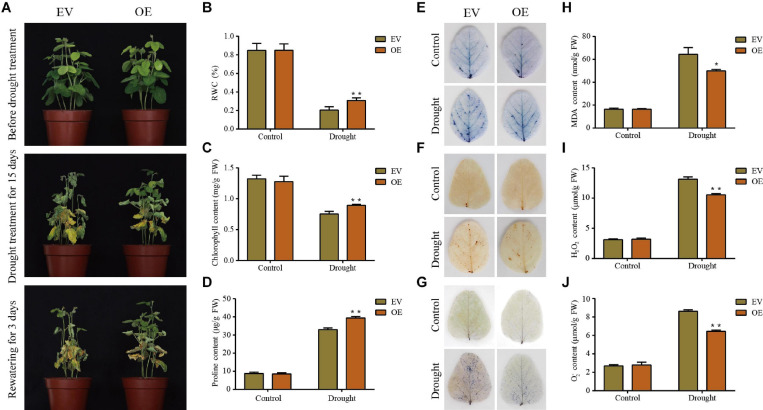
Phenotypic and physiological analyses of the transgenic empty vector (EV) lines and *GmDUF4228-70*-OE (OE) transgenic lines under drought stress. **(A)** Phenotypes of EV and *GmDUF4228-70-*OE transgenic soybean plants treated under drought stress for 15 days and rewatering for 3 days. Relative water content (RWC) **(B)**, chlorophyll content **(C)**, proline content **(D)**, trypan blue staining **(E)** DAB staining **(F)**, NBT staining **(G)**, Malondialdehyde (MDA) content **(H)** H_2_O_2_ content **(I)**, and O^2–^ content **(J)** of the leaves of EV and *GmDUF4228-70-*OE plants grown under drought or control conditions for 7 days. Data are shown as mean ± standard deviation (*n* = 3). Significant differences based on ANOVA are set at *p* < 0.05 (*) and *p* < 0.01 (**), respectively.

**FIGURE 9:**
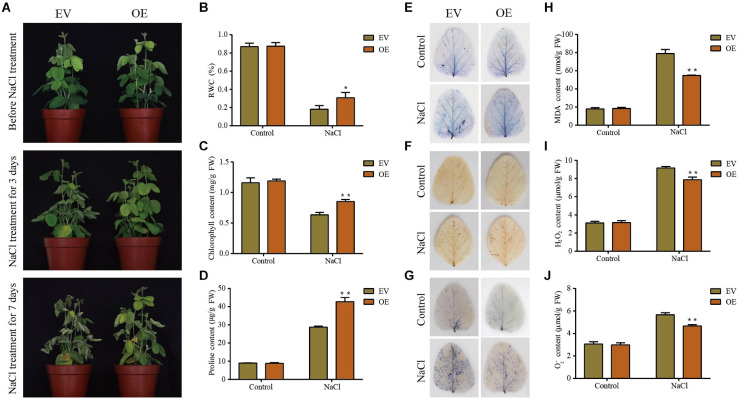
Phenotypic and physiological analyses of the transgenic empty vector (EV) lines and *GmDUF4228-70*-OE (OE) transgenic lines under salt stress. **(A)** Phenotypes of EV and *GmDUF4228-70*-OE transgenic soybean plants treated under salt stress for 3 and 7 days, respectively. Relative water content (RWC) **(B)**, chlorophyll content **(C)**, proline content **(D)**, trypan blue staining **(E)**, DAB staining **(F)**, NBT staining **(G)**, malondialdehyde (MDA) content **(H)**, H_2_O_2_ content **(I)**, and O^2–^ content **(J)** of the leaves of EV and *GmDUF4228-70*-OE plants grown under salt treatment or normal control conditions for 3 days. Data are shown as mean ± standard deviation (*n* = 3). Significant differences based on ANOVA are set at *p* < 0.05 (*) and *p* < 0.01 (**), respectively.

Prior to the treatment with drought or salt, the EV and the *GmDUF4228-70*-OE soybean seedlings showed similar growth phenotypes. Once treated under drought and salt stresses, the *GmDUF4228-70*-OE plants grew better than the EV lines, as indicated by significantly less leaf wilting, curling, and chlorosis (Figure 8A). After 3 days of drought or salt treatment, the EV lines started to show curled and wilted leaves, while the wilting in the *GmDUF4228-70*-OE lines showed trivial change. At the seventh day of salt treatment, all of the leaves in EV lines were wilted, while only some of the leaves in the *GmDUF4228-70*-OE lines became wilted (Figure 9A).

Two physiological indices, i.e., chlorophyll content as an indicator of photosynthetic capacity (Tanaka and Tanaka, 2006) and RWC as an indicator of plant water status, were measured to quantify the effects of salt and drought on the plant development (Meher et al., 2018). The changes in RWC (Figures 8B, 9B) and chlorophyll content (Figures 8C, 9C) of plant leaves in response to drought and salt were consistent with the observation of leaf phenotypes. Specifically, the leaves of *GmDUF4228-70*-OE lines showed a higher RWC and chlorophyll content than those of the EV lines, indicating that the *GmDUF4228-70*-OE lines showed an evident growth advantage compared with the EV lines under drought and salt stresses.

The contents of proline, MDA, H_2_O_2_, and O^2–^ are important indicators used to measure the effects of abiotic stress on plant growth (Wang D. et al., 2019). Proline is a protective agent against osmotic stress, MDA reflects the degree of lipid oxidative damage, while both H_2_O_2_ and O^2–^ play a role of immunity and signal transduction, though excessive accumulation may cause cell membrane damage (Du et al., 2018; Zhang X. Z. et al., 2019). To investigate the underlying physiological mechanisms of *GmDUF4228-70*-OE enhancing plant stress tolerance, we measured the contents of proline, MDA, H_2_O_2_, and O^2–^ in the EV lines and *GmDUF4228-70*-OE lines under normal and stress conditions.

The results showed that under drought and salt stresses, the *GmDUF4228-70*-OE plants showed higher proline contents (Figures 8D, 9D) and lower MDA (Figures 8H, 9H), H_2_O_2_ (Figures 8I, 9I), and O^2–^ (Figures 8J, 9J) contents than the EV lines. In order to visually show the degree of damage in soybean EV and *GmDUF4228-70*-OE plant leaves, we used trypan blue (Figures 8E, 9E), DAB (Figures 8F, 9F), and NBT (Figures 8G, 9G) staining to measure cell viability in soybean leaves under drought and salt stresses. No difference was observed in both the *GmDUF4228-70*-OE and EV soybean leaves under normal conditions based on trypan blue, DAB, or NBT staining methods. However, under the drought and salt treatments, the *GmDUF4228-70*-OE plant leaves were stained significantly less by all three staining methods than the EV plant leaves, suggesting that the leaves of *GmDUF4228-70*-OE plants suffered less damage under drought and salt treatments than the EV plants.

### GmDUF4228-70-OE Plants Exhibited Increased Transcripts of Drought- and Salt-Inducible Genes

**FIGURE 10:**
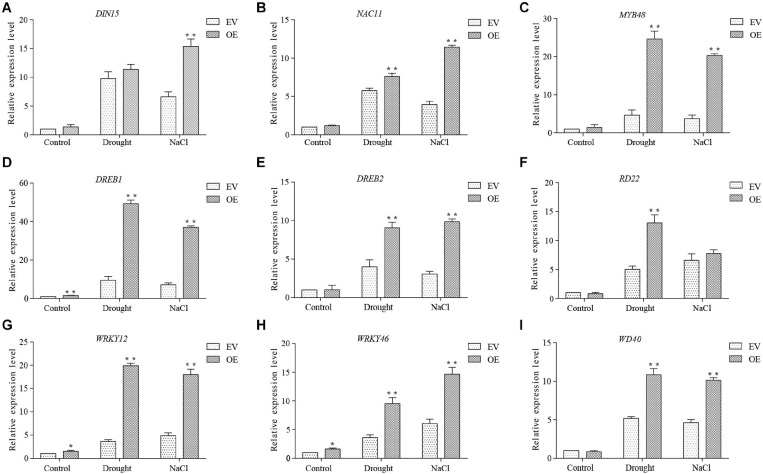
The expression levels of stress-related marker genes *DIN15* **(A)**, *NAC11* **(B)**, *MYB48* **(C)**, *DREB1* **(D)**, *DREB2* **(E)**, *RD22* **(F)**, *WRKY12* **(G)**, *WRKY46* **(H)**, and *WD40* **(I)** based on qRT-PCR. The actin gene is used as an internal control. Data are shown as mean ± standard deviation (*n* = 3). Significant differences based on ANOVA are set at *p* < 0.05 (*) and *p* < 0.01 (**), respectively.

A group of 9 marker genes up-regulated under both drought and salt stresses were chosen based on previous studies to investigate the molecular mechanism of *GmDUF4228-70* in response to stress, including *DIN15* (Guo et al., 2019), *NAC11* (Hao et al., 2011), *MYB48* (Liao et al., 2008), *DREB1* (Kasuga et al., 1999), *DREB2* (Chen et al., 2007), *RD22* (Matus et al., 2014), *WRKY12* (Shi et al., 2018), *WRKY46* (Chen et al., 2017), and *WD40* (Mishra et al., 2014; Figure 10). The expression of these marker genes under normal and stress conditions was analyzed by qRT-PCR. Under normal and stress conditions, the transcript levels of *DREB1*, *WRKY12*, and *WRKY46* in *GmDUF4228-70*-OE were significantly up-regulated compared with those in EV lines, while no significant difference was observed in *DIN15*, *NAC11*, *MYB48*, *DREB2*, *RD22*, and *WD40* under normal conditions. The transcriptional levels of *NAC11*, *MYB48*, *DREB2*, *RD22*, and *WD40* were significantly higher in *GmDUF4228-70*-OE than EV under drought treatment, while the transcriptional levels of *DIN15*, *NAC11*, *MYB48*, *DREB2*, and *WD40* were significantly higher in *GmDUF4228-70*-OE than EV under salt treatment (Figure 10). Under drought and salt treatments, the transcriptional levels of these marker genes increased by varied degrees, indicating that these marker genes may be regulated by *GmDUF4228-70*. Further studies are necessary to identify the relationships between *GmDUF4228-70* and stress-related marker genes in soybean.

## Discussion

DUF4228 has been reported to function in various abiotic stress processes of plants (Wang et al., 2017). However, the role of DUF4228 genes in soybean have not been investigated. Previous studies have identified a total of 22 *DUF4228* genes in *Aquilegia coerulea*, 25 in *Arabidopsis*, 29 in *Medicago truncatula*, and 52 in *Populus trichocarpa* (Yang Q. et al., 2020). Our results of phylogenetic analysis using ML method showed that the topological relationships among a total of 135 DUF4228 protein sequences were largely consistent with those reported previously by Yang Q. et al. (2020) with one exception, i.e., *AT1G18290* identified in Group I of Yang Q. et al. (2020) was revealed in Group II in our study.

Profiling of *GmDUF4228* gene expression showed that most of the genes were expressed low in various tissues and organs of soybean (Figure 4). Our results showed that 27 and 22% of *GmDUF4228* genes were up-regulated under drought and salt stress conditions, respectively (Figure 5), indicating that most of these genes may be related to abiotic stresses. It has been shown that *cis*-acting elements in the promoter region are closely related to gene function (Chow et al., 2018). Consistent with their expression profiles, *GmDUF4228* genes contained a variety of *cis*-acting elements related to abiotic stresses, including MYB, MYC, MBS, and GT-1 elements (Figure 6), which play an important role in response to drought and salt treatments (Abe et al., 1997; Li et al., 2018; Tang et al., 2019; Zhang L. et al., 2019). Studies have shown that MYC also functions in cold stress (Ohta et al., 2018) and disease resistance (Lorenzo et al., 2004). Other elements found in *GmDUF4228* gene promoters are also associated with abiotic stress. For example, the ABRE element is bound by ABRE-binding proteins, involved in dehydration, salt, and ABA stresses (Wang X. et al., 2019), while both ERE and TC-rich repeats may be associated with ethylene and defense and stress response-related elements (Zhang et al., 2015; Song et al., 2019). ARE, LTR, and CGTCA-motif are associated with anaerobic inducement response (Geffers et al., 2001), low temperature stress (Maestrini et al., 2009), and methyl jasmonate response (Xu et al., 2018), respectively. These results suggest that soybean *GmDUF4228* genes may be involved in abiotic stress response.

Soil drought and salinization are two major stress factors influencing plant growth and development. Plants have evolved a variety of survival mechanisms to cope with stress, including the maintenance of morphological characteristics related to plant tissues and physiological mechanisms related to the regulation of metabolism (Vijayaraghavareddy et al., 2020). Under drought stress, in order to improve water use efficiency, stomatal opening on leaves is mainly regulated to control carbon dioxide emissions and reduce transpiration (Mega et al., 2019). In response to drought stress, roots generate CLE25 peptide, which is transferred to leaves via the vascular system to activate NCED3 and promote ABA production (Takahashi et al., 2018). Under salt stress, the growth and development of plants are mainly inhibited by changes in osmotic pressure and ion imbalance. The excessive accumulation of Na^+^ and Cl^–^ in the cell leads to inhibition of the activity of intracellular enzymes involved in Calvin cycle, phenylpropionic acid pathway, glycolysis, and starch synthesis, resulting in disruption of metabolism, ultimately inhibiting plant growth (Zhao et al., 2020). Under salt stress, when roots perceive the change in osmotic pressure, the signal is almost immediately transmitted to the leaves through the stem, causing a decrease in leaf stomatal opening, thus protecting the plant against water loss (Cosgrove and Hedrich, 1991; Christmann et al., 2013). Both drought and salt stresses are perceived by the root system, which transmits a signal to the leaves through a feedback mechanism, causing leaves damaged to varying degrees. Our results showed that the overexpression of *GmDUF4228-70* in soybean reduced water loss and chlorophyll degradation, while improved the viability of soybean under drought and salt stresses. Similarly, the cell activity of *GmDUF4228-70*-OE plant leaves was stronger than that of EV leaves, suggesting that *GmDUF4228* improves the survival of soybean under drought and salt stresses.

In this study, *Agrobacterium rhizogenes*-mediated soybean hairy root transformation technology was used to induce transgenic roots on soybeans to study the function of *GmDUF4228-70* gene (Kereszt et al., 2007). Phenotypic and physiological analyses showed that overexpression of *GmDUF4228-70* improved soybean tolerance to drought and salt (Figures 8, 9). While *Agrobacterium rhizogenes*-mediated transformation of soybean roots generated transgenic seedlings with increased expression of *GmDUF4228-70* gene in roots, this gene cannot be inherited to future generations by sexual reproduction (i.e., seeds). Further transformation studies are necessary to explore the applications of *GmDUF4228-70* gene in breeding of transgenic soybean lines with increased tolerance to drought and salt stresses. In summary, our study provides a scientific foundation for further analysis of the functions of *GmDUF4228-70* gene in response to drought and salt stresses.

## Conclusion

This study identified a total of 81 *DUF4228* genes in the soybean genome. The expression levels of nine of these genes were up-regulated under both drought and salt stresses as identified by the RNA-seq transcriptome data and further validated by qRT-PCR. The results showed that *GmDUF4228-70* was sensitive to both drought and salt stresses. Analysis of plant phenotypes and stress-related physiological indicators showed that overexpression of *GmDUF4228-70* enhanced soybean tolerance to drought and salt stresses. The results of qRT-PCR showed that the transcription levels of marker genes in transgenic soybean overexpressing *GmDUF4228-70* were increased significantly under drought and salt stresses. These results have advanced our understanding of the functions of GmDUF42228 family and provide a strong foundation for further investigations on the molecular mechanisms of *GmDUF4228-70* in response to abiotic stresses in soybean.

## Data Availability Statement

The datasets presented in this study can be found in online repositories. The names of the repository/repositories and accession number(s) can be found in the article/Supplementary Material.

## Author Contributions

Z-SX coordinated the project, conceived and designed experiments, and edited the manuscript. Z-XL performed experiments and wrote the first draft. YL revised the manuscript. Z-YC, JG, JC, and Y-BZ contributed to data analysis and managed reagents. MC, Y-ZM, and X-YC contributed with valuable discussions. All authors reviewed and approved the final manuscript.

## Conflict of Interest

The authors declare that the research was conducted in the absence of any commercial or financial relationships that could be construed as a potential conflict of interest.

## Footnotes

1 http://smart.embl-heidelberg.de/

2 https://www.ncbi.nlm.nih.gov/Structure/cdd/wrpsb.cgi

3 https://www.expasy.org/

4 http://gsds.cbi.pku.edu.cn/

5 http://meme-suite.org/tools/meme

6 https://phytozome.jgi.doe.gov/pz/portal.html

7 http://bioinformatics.psb.ugent.be/webtools/plantcare/html/

## Supplementary Material

Supplementary Figure 1 qRT-PCR analysis of *GmDUF4228-70* expression level in EV and *GmDUF4228-70*-OE transgenic hairy roots.

Supplementary Table 1 Primers and sequences used in this study.

Supplementary Table 2 FPKM values for *DUF4228* genes in different tissues and organs of soybean.

## Abbreviations

CDS: Coding sequence
FPKM: Fragments per kilobase of transcript per million mapped reads
GSDS: Gene structure display server
HMM: Hidden Markov model
MDA: Malondialdehyde
MEME: Multiple Em for motif elicitation
MEGA: Molecular evolutionary genetics analysis
p*I*: Isoelectric point
qRT-PCR: quantitative real-time PCR
RNA-Seq: RNA sequencing
SMART: Simple modular architecture research tool.
