# Supplementary figures and images for: Genome-Wide Analysis of the DUF4228 Family in Soybean and Functional Identification of GmDUF4228–70 in Response to Drought and Salt Stresses
Source: Front Plant Sci. 2021 May 17;12:628299. doi: 10.3389/fpls.2021.628299 (PMC8166234; doi:10.3389/fpls.2021.628299)

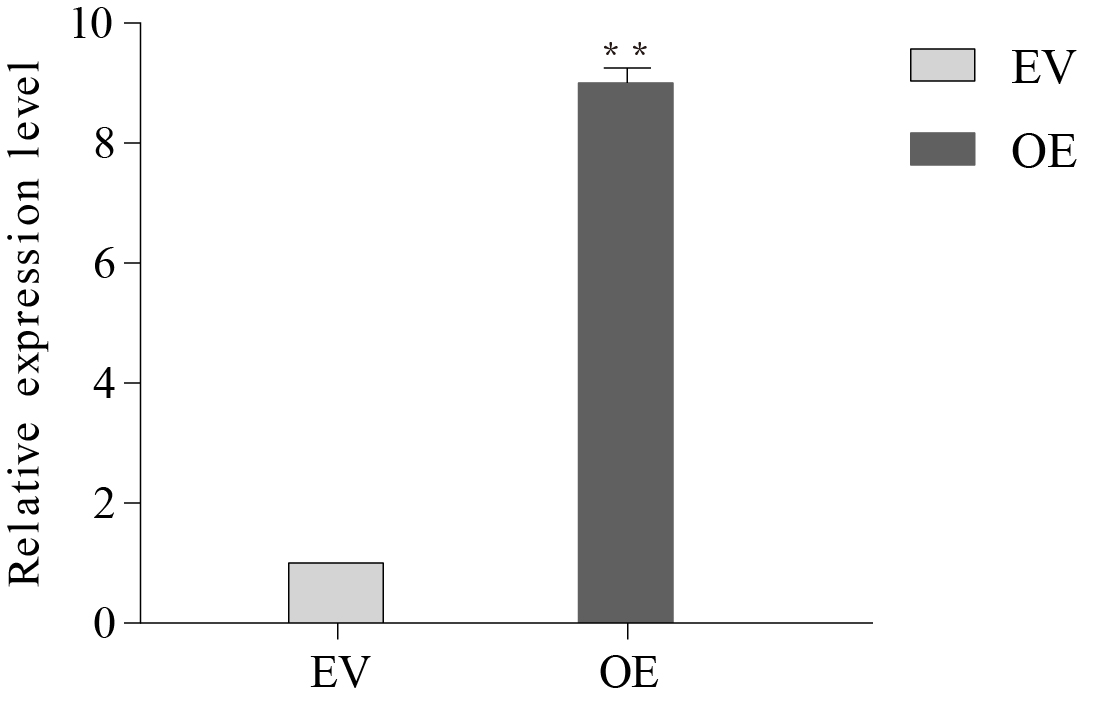

Supplement: Supplementary Figure 1 — qRT-PCR analysis of GmDUF4228-70 expression level in EV and GmDUF4228-70-OE transgenic hairy roots. [file Data_Sheet_1.zip › Figure S1.jpg]
